# 

**Published:** 1877-04

**Authors:** 


### Books and Pamphlets Received.

Annual report of the Supervising Surgeon-General of the Marine Hospital Service of the United States for the fiscal year 1876. John M. Woodworth, M. D., Washington Government Printing Office.

Transactions of the New York Pathological Society, volume I. Based on the proceeding for the year 1875, and largely supplemented from the records of 1844 to 1872. John C. Peters, M. D., editor, 1876. New York: William Wood & Co.

An Elementary Treatise on Diseases of the Skin, for the use of students and practitioners. By Henry G. Piffard, A. M. M. D. London and New York: McMillian & Co., 1876. Buffalo: Herger & Ulbrich.

Contributions to Repairtive Surgery. By Gurdon Buck, M. D. Illustrated. New York: D. Appleton & Co., 1876. Buffalo: Herger & Ulbrich.

Report of the Dedicatory Exercises of the new building of Rush Medical College.

Catalogue and list of graduates of Jefferson Medical College, session of 1876–77.

Salicylic Acid: The experience of Maine physicians in its use. Reported by Frederic Henry Gerrish, M. D. Reprinted from the Transactions of the Maine Medical Association.

A Clinical Lecture on the Treatment of Incipient Stricture by Otis' Operation. Delivered at University College Hospital, London, March 16, 1876, by Mr. Berkeley Hill. Reprinted from the London "Lancet" of April 8, 1876.

An Address on Some of the Leading Public Health Questions; with remarks on the extent of swamp lands in the United States and their reclamation as a sanatary and economic measure. Delivered at the opening of the third annual meeting of the American Public Health Association, Baltimore, Md., November 9, 1875, by J. M. Toner, M. D.

Transactions of the Medical Society of the County of Erie. Semi-annual meeting June 13, 1876. Fifty-sixth annual meeting January 9, 1877. Buffalo: Haas & Klein.

On the Importance of the Uterine Ebb as a Factor in Pelvic Surgery. By Horatio R. Storer, M. D., of Boston, Mass., former Vice-President of the American Medical Association, and member of the Medico-Chirurgical and Obstetrical Societies of Edinburgh. Reprinted from the Edinburgh *Medical Journal* for January, 1877.

The Use of Uterine Supporters. By Clifton E. Wing, M. D., Boston, Mass.

Seventh Annual Report of the New York Opthalmic and Aural Institute, for the year ending December 31, 1876.

Pneumonic Pressure and the Genu-Pectoral Posture in the reduction of uterine laxations. By A. Libley Campbell, M. D., Augusta, Ga. William Wood & Co., New York, 1877.

Annual announcement of the Medical College of the Pacific.

An Illustrated Catalogue of Medical Publications. By William Wood & Co., New York.

The American Medical Association and the Pharmacopœia of the United States of America. By Edward R. Squibb, M. D., Brooklyn, N. Y.

The Untted States Pharmacopœia and the American Medical Association. To be obtained of Dr. H. C. Wood, 1631 Arch street, Philadelphia, by enclosing address and three cent stamp.

### Contents of April Number.

### ORIGINAL.

**Containing Original Articles, Reports of Medical Societies and Hospitals, Editorial, Reviews, Correspondence, Army News, etc., etc ; including the usual variety of Medical Periodical Publications. Specimen copies sent on application.**

Address Buffalo Medical & Surgical Journal, Buffalo, N. Y.

TERMS OF SUBSCRIPTION, $3.00 PER YEAR, IN ADVANCE.

